# Enhanced maximum intensity projection (eMIP) for improving the fidelity of optoacoustic images

**DOI:** 10.1038/s44303-025-00112-z

**Published:** 2025-10-06

**Authors:** Manuel Gehmeyr, María Begoña Rojas López, Suhanyaa Nitkunanantharajah, Hubert Preißl, Andreas Vosseler, Reiner Jumpertz von Schwartzenberg, Andreas L. Birkenfeld, Nikoletta Katsouli, Nikolina-Alexia Fasoula, Angelos Karlas, Michael Kallmayer, Anette-Gabriele Ziegler, Dominik Jüstel, Vasilis Ntziachristos

**Affiliations:** 1 Chair of Biological Imaging, Central Institute for Translational Cancer Research (TranslaTUM), School of Medicine and Health & School of Computation, Information and Technology, Technical University of Munich, Munich, Germany; 2 Institute of Biological and Medical Imaging, Bioengineering Center, Helmholtz Zentrum München, Neuherberg, Germany; 3 Institute of Computational Biology, Helmholtz Zentrum München, Neuherberg, Germany; 4 German Center for Diabetes Research (DZD), Neuherberg, Germany; 5 Department of Diabetology, Endocrinology and Nephrology, Eberhard-Karls University Tübingen, Tübingen, Germany; 6 Institute for Diabetes Research and Metabolic Diseases (IDM) of the Helmholtz Center Munich at the University of Tübingen, Tübingen, Germany; 7 Institute of Pharmaceutical Sciences, Department of Pharmacy and Biochemistry, Eberhard-Karls University Tübingen, Tübingen, Germany; 8 Cluster of Excellence EXC 2124 Controlling Microbes to Fight Infections, University of Tübingen, Tübingen, Germany; 9 M3 Research Center, Malignome, Metabolome and Microbiome, Tübingen, Germany; 10 Diabetes & Obesity Theme, School of Cardiovascular and Metabolic Medicine & Sciences, Faculty of Life Sciences & Medicine, Kings College London, London, UK; 11 Clinic and Polyclinic for Vascular and Endovascular Surgery, TUM University Hospital, Hospital rechts der Isar, Technical University of Munich, Munich, Germany; 12 German Centre for Cardiovascular Research (DZHK), Partner Site Munich Heart Alliance, Munich, Germany; 13 Chair for Computer Aided Medical Procedures & Augmented Reality, Technical University of Munich, Munich, Germany; 14 Institute of Diabetes Research, German Research Center for Environmental Health, Helmholtz Zentrum München, Munich-Neuherberg, Germany; 15 Forschergruppe Diabetes, Technical University Munich, Klinikum Rechts Der Isar, Munich, Germany; 16 Munich Institute of Robotics and Machine Intelligence (MIRMI), Technical University of Munich, Munich, Germany

**Keywords:** Optics and photonics, Medical imaging

## Abstract

Three-dimensional (3D) image reconstructions are often rendered as two-dimensional images, using maximum intensity projections (MIPs). However, MIP’s rendering fidelity depends on the alignment of the individual slices along the projection direction. Also, the presence of noise and artifacts affects the contrast and the projected image elements. We introduce enhanced MIP (eMIP), a methodology that aligns the boundaries (e.g., skin boundary) of adjacent slices of the 3D volume onto the same coordinate system assumed by MIP (e.g., same depth) and applies robust contrast adjustment to normalize the intensities of the projected slices. We benchmark eMIP on 1725 clinical scans of human skin, using raster-scan optoacoustic mesoscopy (RSOM) that were assessed by 8 experts. Our results show that eMIP facilitates interpretability compared to conventional MIP and increases consistently the perceived image quality. The improved diagnostic ability of eMIP has the potential to replace MIP in RSOM and similar modalities.

## Introduction

The maximum intensity projection (MIP) is a useful method to render three-dimensional (3D) slices of a volumetric reconstruction as a two-dimensional (2D) image. It is based on projecting voxels with high intensity from each 3D slice onto the same 2D plane, thus recording the features of highest contrast observed in the reconstructed volume. MIP is utilized in different medical imaging applications, including magnetic resonance angiography, as well as radiological or nuclear imaging studies^1–5^ to enhance the visibility of high-density structures.

To exemplify MIP operation, we describe MIP usage in the context of raster-scan optoacoustic mesoscopy (RSOM), an emerging modality that allows non-invasive volumetric imaging of the skin and dermal microvasculature, with resolution in the few tens of micrometers or better^6–8^. RSOM’s promising potential to diagnose and monitor cardiometabolic and skin diseases^9–13^ requires high-fidelity representations of the 3D reconstructed tissue volume. However, the accuracy of MIP-generated 2D representations of RSOM volumetric reconstructions is affected by image artifacts (e.g., strong contrast from hairs, artifacts from the reconstruction & frequency filtering, reflections in the transducer, or shot noise), which produce random volume elements of high intensity and misguide the MIP registration^14^. Another challenge relates to the relative positioning of the 3D slices and tissue boundaries (e.g., the skin surface) in relation to the coordinate system used for computing the maximum intensity projections. For example, when the MIP method is applied to skin RSOM images, a poor relative positioning of the skin boundary with the scanning directions will result in overlapping of skin and vascular structures, challenging image interpretation and stymying the use of RSOM diagnostically and theranostically^15,16^.

Therefore, we aimed to develop a data pre-processing methodology that can align the 3D skin boundary for consistent visualization of MIP and reduce the influence of image artifacts to increase the reliability and accuracy of the projections. Termed enhanced-MIP (eMIP), our method first estimates robustly the skin surface in the 3D volume to posteriorly align the scan with the lateral projection directions employed by MIP. The skin surface is identified based on detecting the melanin (a highly absorbing natural pigment that provides color to the skin, eyes, and hair) in the stratum-corneum, the outermost layer of the epidermis. The 3D RSOM volume is aligned or flattened by shifting voxel columns in the 3D array along the depth axis, so that the skin boundary is positioned on a plane parallel to the lateral projection directions. Previously, some motion correction algorithms have also used the melanin layer to detect the skin surface on RSOM data^8^ and for skin flattening^12,17^, but these algorithms employed manual anatomical segmentation or user-based tuning of algorithmic parameters^18^. Conversely, a major eMIP focus was the development of automated processes that do not require user intervention. Furthermore, previous algorithms were sensitive to surface discontinuations, such as hair. Therefore, a second eMIP goal was to identify artefacts and discontinuities in the image and remove erroneous high-intensity absorbers, so that the skin surface model is estimated reliably for subsequent alignment on the MIP coordinate system. eMIP aims to achieve these objectives through iterative polynomial fits to robustly estimate the position of the skin surface and outlier exclusions.

Another aspect in order to ensure image fidelity was to improve the visualization of the reconstructed volume in the context of frequency band equalization (FBE)^11^ employed in RSOM images. FBE differentially processes high and low frequency signals in the broadband (∼10–100 MHz) collected by RSOM^12,19–22^. Using digital signal low-pass and high-pass filtering operations, FBE separates the optoacoustic signals into two frequency bands, so that two 3D volumes are reconstructed^6^. This approach enhances the visibility of small structures associated with high frequency components that could be imperceptible if rendered in the same color and intensity scale as the much higher-intensity low frequency signals. Finally, MIPs are taken along the desired direction and fused to create a composite image, in which images reconstructed in the high frequency band are rendered in a different color than images of the low frequency band.

In this work, we improve the scaling method used by FBE for the composite image to ensure that the contrast rendered refers to voxels corresponding to true high-contrast tissue structures in both frequency channels. eMIP was evaluated on a set of 1725 RSOM scans taken on the forearm and leg of human participants. All images obtained by eMIP or MIP^11^ were assessed by eight experts in a random order and each image was assigned a score (on a scale from 1 to 4 with 4 being the best score) indicating visual quality. The 3450 eMIP and MIP images constitute the first comprehensive set of quality-assessed RSOM images that allowed eMIP to be benchmarked against MIP. For each scan, we average the rates and compute the mean opinion score (MOS) for comparison of the two methods, since MOSs are a more reliable quantification metric than individual ratings. Using the MOS dataset, we show that eMIP improved significantly the perceived image quality of the RSOM scans. Finally, the comprehensive dataset itself can be used in the future as a starting point to develop faster quality assessment methods for RSOM images. In summary, eMIP effectively addresses the limitations of conventional MIP by realigning slice boundaries and employing advanced contrast normalization, resulting in more accurate 2D renderings and enhanced diagnostic potential for RSOM and possibly a variety of 3D medical imaging modalities.

## Results

### Limitation of MIP for imaging RSOM volumes with tilted skin

**Fig. 1:**
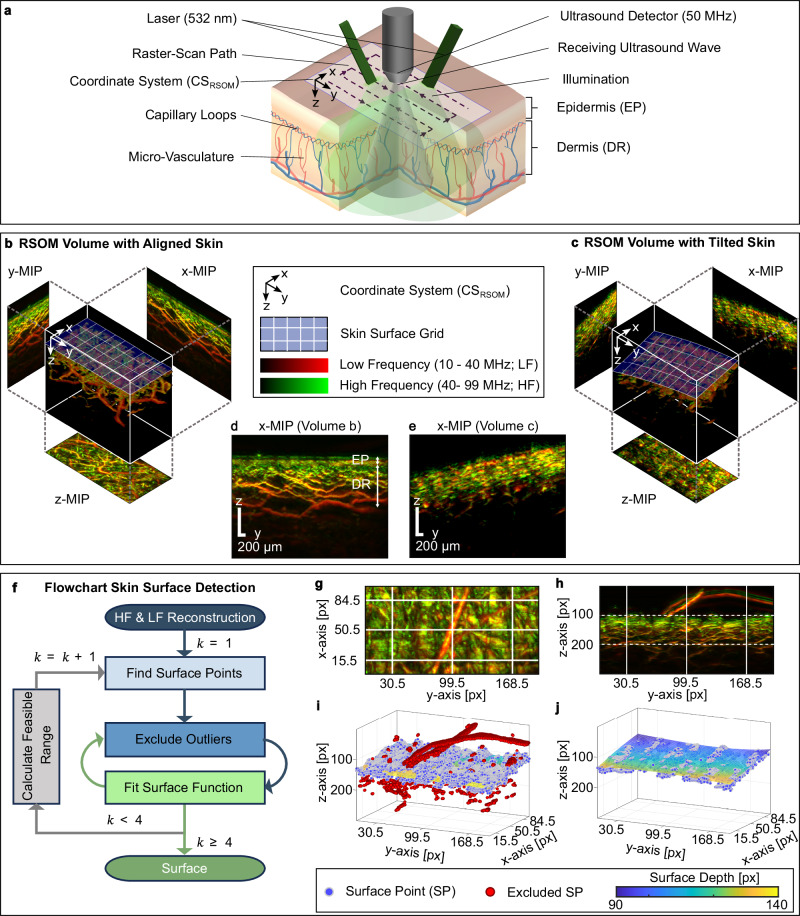
Our developed method enhanced maximum intensity projection (eMIP) that improves the maximum intensity projection (MIP) of raster-scan optoacoustic mesoscopy (RSOM) scans by correcting for misalignments of the skin. **a** Schematic depicting the RSOM scanning process on skin. The RSOM coordinate system (CS_RSOM_) is shown in a corner of the scanned region. **b**, **c** Rendered RSOM reconstructed volumes and corresponding maximum intensity projections (MIP) along the three coordinate axes, defined by the CS_RSOM_. The volumes were obtained after separately reconstructing raw data of two frequency bands (low and high frequency, LF and HF, respectively), and merging the reconstructions. The LF reconstruction, in red, indicates larger vessels and HF reconstruction, in green, indicates smaller vascular structures. The skin surface is represented with a blue grid in the volumetric reconstruction. **d**, **e** Lateral MIPs along the $x$-direction ($x$-MIPs) corresponding to the volumes in (**b**) and (**c**), respectively. As the skin surface is a plane orthogonal to the z-axis in (**b**), in the lateral MIP, the epidermis (EP) and dermis (DR) are clearly resolved thanks to their characteristic vascular morphology. In contrast, when the skin surface is not parallel to the $xy$ -plane of CS_RSOM_ (as in the volume **c**), the different vascular structures overlap in the lateral direction, making it challenging to distinguish between the skin layers. **f** Skin surface detection workflow. The inputs were LF and HF reconstructed RSOM volumes. The output was a polynomial surface function. The workflow consists of four building blocks: “Find Surface Points”, “Exclude Outliers”, “Fit Surface Function” and “Calculate Feasible Range”, that were run in iterative loops (iterations denoted by *k*). **g**–**j** Skin surface detection illustrated using a representative scan with two hairs. **g** Division of raster tiles for surface point detection shown on the z-MIP (see Methods; lateral dimensions: 99×198 pixels (px)). **h** $x$ -MIP of the reconstruction with two visible hairs. **i** 3D plot of surface points (blue) and fitted surface function after the first (outer loop) iteration (Flowchart **f**). Outlier voxels are indicated in red (from the two hairs above the surface and vessels below the surface). **j** 3D plot of the final surface function with fitted surface points colored by depth. No outliers needed to be excluded, due to a narrow search range for surface points in the last (fourth) iteration.

RSOM detects ultrasound waves after optical excitation of chromophores at predefined points along a raster pattern on the surface of the tissue of interest. Figure 1a shows the RSOM trajectory by a dashed line inside the scanning area, which defines in combination with the perpendicular scanning direction (depth) the coordinate system of the RSOM scan (CS_RSOM_, see Methods). After scanning, the optoacoustic recorded signal is separated into two frequency bands with a low frequency band (LF; 10–40 MHz) and a high frequency band (HF; 40–99 MHz) bandpass filters and independently reconstructed producing two 3D-volumes. The two volumes, i.e., the LF and HF reconstruction, are then merged using different color channels (e.g., Fig. 1b, c). This frequency separation allows large blood vessels (LF signals) to be colored in red and the small vascular structures (HF signals) to be colored in green in a combined RGB-image, where small structures get enhanced according to FBE (see Methods).

As medical doctors make their diagnosis based on 2D images, 3D volumes need to be rendered into 2D images. The MIP method (see Methods) can generate 2D images by projecting the maximum intensity values of the 3D slices along the chosen direction ($x$, $y$ or $z$; Fig. 1b, c) onto the projection plane. Figure 1d, e shows the resulting lateral MIPs from the volumes represented in Fig. 1b, c, respectively, to illustrate the effect of an aligned skin surface on the 2D MIP-generated image. When the skin is positioned with respect to CS_RSOM_ such that the skin surface is parallel to the ${xy}$ plane, like the 3D volume in Fig. 1b, the skin morphology is easily identifiable in the $x$-MIP or $y$-MIP, due to the different cutaneous vasculature. Thus, we could clearly mark in the $x$ -MIP in Fig. 1d the epidermis (EP) – determined by the melanin layer – and dermis (DR) – characterized by the vascular networks in the capillary loops and the vascular plexus. However, when the skin is tilted (for example, due to a misaligned ultrasound detector) or exhibits curvature (as from applied pressure), the limitation of MIP becomes apparent. In these situations, the epidermis and dermis are at different depths along the ${yz}$ (or $xz$) plane (Fig. 1c), meaning that vascular structures belonging to different layers overlap throughout the *x*-MIP (Fig. 1e), making it challenging to distinguish the epidermis from the dermis and visualize the vasculature clearly.

### The eMIP method consistently renders representative 2D images

To overcome the afore-mentioned limitation of MIP, we developed eMIP to improve the 2D visualization of 3D volumes (see Methods). The eMIP method has an algorithm that automatically corrects the curvature and tilt of the skin surface on a 3D volume, so that the corrected skin surface is shifted to be parallel to the ${xy}$ plane at a defined $z$ value (i.e., depth). Figure 1f illustrates eMIP’s workflow to achieve the skin flattening. Its surface detection method first approximates the skin surface in the 3D reconstructed volume using a polynomial model fitted through a linear least squares subroutine. Next, outliers are iteratively excluded. The outlier exclusion is essential to disregard hairs, noise or other high absorbers that are not part of the skin, such as shown in the representative RSOM images in Fig. 1g, h. Figure 1i, j demonstrates how eMIP can successfully identify hairs as outliers (red), so that a suitable polynomial that approximates the skin surface can be chosen (Fig. 1j).

### Images rendered by eMIP are higher quality than images rendered by MIP

**Fig. 2:**
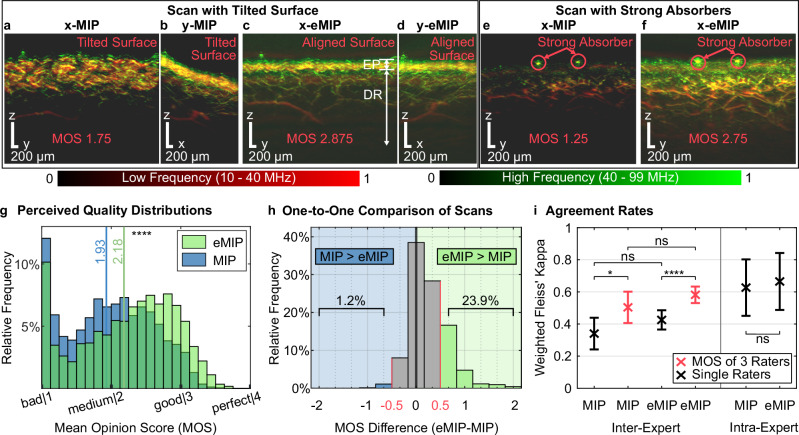
Qualitative and quantitative comparison of our visualization tool for enhanced maximum intensity projection (eMIP) to conventional maximum intensity projection (MIP) for raster-scan optoacoustic mesoscopy (RSOM) images. **a**–**d** Comparing visualizations of an RSOM scan with tilted skin structure along lateral directions generated by conventional MIP (**a**, **b**) and eMIP (**c**, **d**). In the projection along $x$ -direction generated by MIP (**a**), the morphology of the microvasculature cannot be distinguished, because the skin surface (**b**) is not aligned with the RSOM coordinate system (CS_RSOM_). After surface alignment using eMIP, the resulting RSOM image projected along the $x$-direction (**c**) depicts the skin layers, marked in the image as EP for epidermis and DR for dermis. The maximum intensity projection along $y$ (**d**) shows a straight skin line. The mean opinion score (MOS) is indicated in each RSOM image. **e, f** eMIP enhances the visualization of the scan with strong absorbers compared to MIP (**e**), as eMIP (**f**) includes improved contrast adjustment. **g** Histogram of the averaged image quality MOSs for both visualization techniques (*n* = 1725 scans, each assessed by 8 experts). Mean values are indicated with vertical lines. **h** Histogram showing differences in MOSs between eMIP and MIP for 1725 individually assessed images. 23.9% of scans visualized by eMIP show an increase of at least 0.5 MOS points compared to MIP, while only 1.2% of the scans visualized by eMIP were deemed to be 0.5 MOS point lower than MIP. **i** Inter- and intra- expert agreement on perceived image quality ratings for MIP, eMIP. In black the comparison between single raters and in red the comparison between groups of 3 raters. Error bars indicate 95% confidence intervals. Statistical analysis by right-tailed two-sample *t*-test (**g**) and according to the tests defined in Methods (**i**), ns: *p* >0.05, *p ≤0.05, ****p ≤0.0001.

Figure 2 highlights the improvements in quality achieved by eMIP over the reference method MIP, both qualitatively using representative images and quantitatively through quality scores derived from expert assessment. Figure 2a–d demonstrates the benefits of eMIP’s skin surface correction when the skin is skewed in the 3D volume along one of the side projections. As explained above, eMIP visualization aims to solve the problem of a tilted and curved skin surface with respect to conventional MIP.

Furthermore, integrating dynamic contrast adjustment through FBE into the eMIP enables deeper, more accurate and detailed visualization of skin microvasculature compared to the established techniques applied to MIP (see Methods). Figure 2e, f present 2D projections along the $x$-direction using MIP and eMIP. The overall brightness of $x$-MIP in Fig. 2e is low due to naïve linear contrast scaling, with maximum reference intensity set by strong absorbers (marked by red circles). The image of the same RSOM scan displayed by eMIP (Fig. 2f) after adjusting the contrast dynamically, showcases well-adjusted brightness and a clearer visualization of the deeper dermal microvasculature.

More examples of RSOM scans visualized using both methods can be found in Supplementary Fig. 1 (including rare cases where the surface detection failed Supplementary Fig. 1b, c and where MIP was preferred over eMIP due to higher contrast Supplementary Fig. 1d).

The MOS (mean opinion score as defined in Methods), was analyzed to quantitatively determine the degree of improvement in image quality when eMIP was compared to MIP. The scores (bad (1), medium (2), good (3) and perfect (4)) were assigned by eight experts (Methods), who assessed the quality of 1725 RSOM scans visualized using both MIP and eMIP. For the two representative scans in Fig. 2a–f, improvement in perceived image quality was confirmed quantitatively by a MOS increase of 1.5 and 1.035 points, respectively.

This improvement in perceived image quality is likewise observed across the entire dataset of 1725 scans. Figure 2g exhibits the mean and distribution of the MOS from both visualization techniques. The distribution corresponding to eMIP shows a general right shift towards higher quality with a lower proportion of images labeled as “bad” quality. The average MOS of eMIP-rendered images increased by 0.250 points ($p\le 0.0001,{CI}=[0.214,\,0.287]$) and median MOS by 0.313 points ($p\le 0.0001,{CI}=[\mathrm{0.250,0.438}]$) compared to MIP-rendered images (see Methods). Qualitatively, the MOS improvement of eMIP over MIP (though varying in magnitude) is consistent across all demographic and technical subgroups we have tested, including skin tone, age, body mass index (BMI), gender, anatomical scan location, baseline quality and relative brightness between eMIP and MIP visualization. For a detailed breakdown see Supplementary Note 1 and Supplementary Table 1.

Furthermore, eMIP method reduced by almost 30% the low quality assessed images (i.e., out of the total images, those with a score of less than 1.5 MOS points decreased from 26.0% with MIP to 18.4% with eMIP).

When images generated by MIP and eMIP were compared on a one-to-one basis for each scan (see Methods), eMIP achieved MOSs at least 0.5 points higher than MIP in 23.9% of the 1725 scans. In contrast, only 1.2% of the eMIP images were rated with MOSs at least 0.5 points lower than their corresponding MIP images (potentially due to failure in the surface detection). The one-to-one comparison is illustrated in the histogram of Fig. 2d, which highlights the quality improvements offered by our method compared to the reference, revealing systematic weaknesses in the MIP image formation process (e.g., dark or superimposed images). These findings are based on the assumptions that a difference of 0.5 MOS points corresponds to a substantial quality improvement, likely attributed to suboptimal visualization in the lower-quality image.

### Improvements in perceptual image quality assessment (IQA)

In the previous section, we used perceptual IQA, i.e., the MOS of the 8 experts, as the metric for image quality to facilitate a quantitative comparison of the two visualization methods. Additionally, perceptual IQA is an important tool for data curation, but it inherently involves a degree of subjectivity. Therefore, achieving high consistency in ratings is essential for meaningful results. This can be facilitated by ensuring that image quality is straightforward to interpret by the raters or by aggregating the MOS from multiple raters, leveraging the effect of regression toward the mean. A common metric to measure the consistency is the (weighted) Fleiss’ kappa for inter-expert (between experts) agreement and intra-expert (within a single expert) agreement (see Methods).

Figure 2i illustrates the inter- and intra-expert agreement coefficient on the images of MIP and eMIP. The figure shows the agreement coefficient comparing single raters (shown in black) and the average agreement when comparing the MOS of groups of three raters (shown in red). Table 1 provides an interpretation of the agreement coefficients.

**Table 1:** Interpretation of agreement coefficient ***κ.***

*κ*	Interpretation
≤0	Poor agreement
0.00–0.20	Slight agreement
0.20–0.40	Fair agreement
0.40–0.60	Moderate agreement
0.60–0.80	Substantial agreement
0.80–1.00	Almost perfect agreement

As expected, intra-expert agreement is higher than inter-expert agreement, although it remains imperfect. Additionally, the fact that inter-expert agreement is only fair to moderate underscores the challenges involved in assessing the image quality of RSOM scans.

However, for both visualization methods, averaging the quality scores from just three raters significantly improves agreement, demonstrating the positive aggregation impact of MOS on reliability.

Furthermore, while not statistically significant (*p*-values: 0.071 (inter), 0.38 (intra), 0.080 (group of 3)), there is a trend suggesting that eMIP images achieve higher agreement rates among the raters compared to MIP images. This implies that eMIP might facilitate more consistent assessment of image quality and that fewer experts are required to achieve comparable reliability.

### Computational cost analysis

Both visualization methods eMIP and MIP are computational efficient, requiring less than a second for the visualization alone (excluding loading and saving) on our test machine (see Methods). On average, eMIP takes 0.98 s and MIP only 0.03 s. Table 2 provides a detailed breakdown of both visualization pipelines alongside the reconstruction time for context.

**Table 2:** Computational times (in seconds) for individual components of the visualization process.

		Mean	STD	Median	Maximum
Both	Load LFR & HFR	0.2853	0.0720	0.2828	1.6246
eMIP	Surface detection	0.8652	0.2518	0.8245	4.4892
	Visualization	0.1120	0.0257	0.1099	0.5248
	Save 2D images	2.4603	1.3505	1.6898	8.5321
	Save surface info	0.0017	0.0006	0.0016	0.0083
	Total	3.7245	1.4452	2.9301	14.8880
MIP	Visualization	0.0309	0.0084	0.0298	0.1520
	Save 2D images	2.3204	1.3569	1.5489	7.8309
	Total	2.6366	1.3693	1.8663	9.5377
Reconstruction LFR & HFR		5.1123	0.7576	5.2151	5.7994

The table provides a detailed breakdown of runtime for each step in MIP & eMIP. Load LFR & HFR times are shared across both techniques. For eMIP, the breakdown includes additionally surface detection, visualization (comprising surface correction & dynamic contrast adjustment), and saving images and surface function to disk. For MIP, the breakdown includes visualization and saving the images to disk. The reconstruction time was collected separately on an optimized workstation without further breakdown. It includes loading, pre-processing (e.g., bandpass filter), 3D image reconstruction and saving to disk. For all durations we show the mean, standard deviation (std), median and maximum over all 1725 scans.

Considering loading and saving image data and surface function to disk both visualization times remain fast enough for real-time use and are suitable for large scale clinical bath processing. The average time for the full MIP visualization is 2.64 s, while eMIP averages 3.72 s, adding a mean overhead of 1.08 s to the total processing time. Notably, a substantial portion of the total time in both pipelines is spent on saving image data due to non-optimized MATLAB routines.

While the surface detection accounts for most of the additional processing time (0.87 s on average) it remains computationally efficient for the given task. Moreover, the surface function can be saved and reused for further visualizations or precomputed in advance, and even in the worst-case, the maximum observed surface detection time remains modest at 4.49 s.

For comparison, the mean reconstruction time for both LF reconstruction (LFR) and HF reconstruction (HFR) is 5.11 s on a highly optimized workstation using GPU-acceleration, illustrating that the overhead introduced by eMIP to the full reconstruction and visualization pipeline is moderate at 14% (7.75 s with MIP vs. 8.84 s with eMIP).

## Discussion

In this work, we introduce a novel image visualization tool for volumetric reconstructions, eMIP, that yields high fidelity 2D representations with respect to the volume. Using a large dataset of RSOM scans, we also demonstrate that, compared to the prevailing visualization method (MIP), eMIP significantly improves the perceived quality of RSOM images due to its ability to compensate for non-ideal positioning of the ultrasound detector when scanning and its improved contrast. Based on this result, eMIP could be included as a post-processing task for medical applications based on projecting 3D volumes into 2D images^1–5^, as it facilitates image interpretation.

The ability of eMIP to automatically detect and flatten the skin surface on 3D scans acquired with imprecisely positioned ultrasound detectors improves the visualization of the 2D projected images. Detecting the skin boundary in RSOM scans and flattening it (i.e., shifting slices of the 3D volume such that the detected skin boundary aligns to a uniform reference level parallel to the ${xy}$ plane) have been used in previous studies as a preprocessing step since the skin surface can act as an anchor for subsequent skin segmentation tasks^12,17,23^. However, the employed algorithm for the alignment had the original purpose of correcting motion and its capacity to flatten the data has not been evaluated in a large dataset, conversely to what eMIP has demonstrated in this work. eMIP can thus be applied as a data homogenization technique previous to data-driven analysis methods for RSOM and other medical image modalities (such as CT angiography^24^) and processes (such as registration of non-static^24^ images). In this context, the standardized alignment and enhanced visibility achieved with eMIP may support posterior analysis of RSOM clinical data by improving the detectability of anatomical biomarkers – such as the epidermal layer or cutaneous microvasculature – and reducing variability across measurements. These improvements could benefit both manual and automated pipelines, particularly for vascular biomarker extraction or longitudinal studies, advancing the development of clinical applications of RSOM. For instance, the authors believe that artificial intelligence (AI) applications could benefit from eMIP-rendered images, reducing the amount of data required to train when all the images show the skin boundary at the same depths and the relevant image content is better visualized (i.e., contrast enhanced).

A current limitation of eMIP, however, is that the hyperparameters (Supplementary Tables 2–5) of the surface detection algorithm were optimized on a limited amount of data (a subset of the dataset of 1725 scans) and conditions (healthy volunteers and patients with cardiovascular diseases or diabetes only). While the set of parameters yielded a consistent quality improvement on the whole dataset of 1725 scans (as demonstrated in this manuscript and supported by the confounder analysis), our surface detection algorithm might need adjustments in other conditions, such as psoriatic skin, where thickened epidermis and sometimes elongated capillary loops^11^ may alter the detection conditions. A reproducibility study on an even larger dataset with more disease conditions, possibly with extended hyperparameter tuning, might further improve eMIP.

To facilitate the adaptation and utilization of eMIP, we implemented eMIP in a toolbox comprising its building blocks (e.g., surface detection and correction), along with detailed documentation. Notably, eMIP can be deactivated or adjusted depending on the clinical use case. For example, in conditions where natural curvature of the skin is diagnostically meaningful – such as in melanoma or edema – flattening might obscure relevant features. In such scenarios, it is possible to either skip the correction step or apply a more conservative flattening strategy (e.g., based on a linear polynomial fit) to remove global tilts while preserving local deformations. To support flexible adaptation, the toolbox exposes a simple sensitivity parameter that governs the stringency of surface point detection, alongside to more advanced hyperparameter such as thresholds for outlier exclusion and the polynomial fitting degree. This design enables users to re-tune the surface detection algorithm for atypical skin morphologies or data where the default configuration fails to generalize. Moreover, the toolbox allows for the export of both the raw and corrected MIPs and could be extended to include visual overlays of the deformation field to ensure transparency and support subsequent analysis.

Additionally, the shift towards higher perceived quality of eMIP-generated images in comparison with MIP-generated images hints how an improper visualization biases data quality assessment^25,26^. eMIP reduced such visualization bias thanks to its increased fidelity with the corresponding volumes, which could prevent experts from erroneously discarding low-quality scans even though the underlying recorded data is of high-quality. Therefore, with this work we demonstrated that eMIP narrows the assessment gap between actual data quality and visual 2D image quality.

The extensive dataset of RSOM scans (*N* = 1725) that we collected to validate our method contains annotations by eight experts with perceived quality scores for every MIP- or eMIP- rendered 2D image. The use of MOS addresses additionally a typical drawback of subjective image quality assessment, by reducing the variability of the individual quality labels^25,27^. Analogous datasets have previously been presented for other modalities^28–30^, but not for RSOM. As such, the collection of this RSOM dataset with subjective quality labels is of great importance for the development of further data-driven RSOM applications or to validate automated, cost-efficient quality assessment methods. These may include mathematically defined objective quality metrics^31^, as well as machine learning-based approaches^32^, which can complement or eventually replace subjective expert scoring in large-scale studies.

The presented eMIP method could be incorporated as part of the image formation step in the RSOM (and other medical image modalities) pipeline, in order to ensure higher image quality in the 2D visualization of a volumetric reconstruction. Incorporating eMIP into the pipeline will benefit both, data analysts and clinicians. While the former could improve the performance of their data-driven algorithms due to a standardized input data; the latter could interpret more confidently medical readings due to anatomically more accurate images.

Even though a higher image quality is preferred for accurate diagnosis^25^, it is worth noting that our visualization method was evaluated in terms of visual quality rather than in terms of diagnostic performance. Incorporating assessments of clinical end-users, as done in^33^, could provide valuable insights into the clinical significance of the eMIP-generated images and help quantify the impact of image distortions on downstream diagnostic metrics, such as disease classification or vessel quantification^34^.

Alternatively to eMIP, volume rendering algorithms and emerging virtual reality visualization methods have been shown to preserve better than maximum intensity projections the 3D relationships of the vessel networks in CT angiographs^35–37^, so a possible line of investigation could study if such improvement is also noticeable for RSOM images.

In summary, we present a visualization tool for 3D medical reconstructed scans, eMIP, and validate it with RSOM scans, showing an improved quality and higher reliability of rendered 2D images, that can be used as a standardization method for subsequent analysis. The integration of eMIP into RSOM could accelerate the widespread clinical adoption of the modality and spur the development of new RSOM applications in the clinic.

## Methods

### Assembly of the RSOM dataset for benchmarking eMIP against MIP

The data used for this manuscript were collected as part of three clinical studies which investigated the effects of diabetes and cardiovascular diseases on skin microvasculature. The studies were run by the University Hospital Klinikum rechts der Isar from Technical University of Munich and Tübingen University Hospital (Germany). Measurements were performed with two RSOM Explorer C50® research systems (iThera Medical GmbH, Germany). In total, 1725 RSOM scans were collected from 236 participants (*N* = 174 subjects recruited in Munich and *N* = 62 subjects in Tübingen). Scans were taken from the inner forearm during cuff occlusion and release (between 7 and 13 scans per participant) and from the pretibial region of the lower leg (between 1 and 3 scans per participant).

The recruited participants in University Hospital Klinikum rechts der Isar from Technical University of Munich^38^ were asked to avoid exercise for 24 h and fast for 6 h prior to the scan. The day of the examination, they were first sitting in a relaxed position for 15 min before the actual examination began. The scan took place in a quiet, semi-dark examination room with normal room temperature (≈23 °C). The recruited participants in Tübingen University Hospital were also asked to avoid exercise for 24 h prior to the examination but they were not in fasting state. On the measurement day, the participants were placed in a quiet room at normal room temperature (≈23 °C), and after they laid for 15 min until the beginning of the measurement, they were scanned in lying position.

Raw RSOM data consists of temporal pressure signals (also known as A-lines) that are generated after illuminating tissue with a monochromatic pulsed laser by means of two customized fiber optic cables (532 nm, 1000 Hz pulse repetition rate, 80 µJ pulse energy). The A-lines were recorded by a single spherically focused LiNbO_3_ ultrasound transducer (50 MHz central frequency, 10–99 MHz bandwidth, 4 mm focal distance) with a sampling frequency of 500 MHz. The device was positioned with the help of mechanical stages in steps of 0.020 mm, following a raster trajectory inside a field of view of 4 mm × 2 mm. A schematic representation of the RSOM device on a skin section is shown in Fig. 1a. The sinogram was then produced by stacking signals over time in the spatial scanning dimensions. Sinograms are made up of multiple B-planes, which consist of all the signals recorded along a single line (fast-scanning axis) during the raster scan. The sinograms were reconstructed using a delay-and-sum algorithm presented in ref. ^6^, which produced volumetric reconstructions of the dermal microvasculature with a voxel size of 20 µm × 20 µm × 4 µm. The scanning process determines the cartesian coordinate system of the RSOM scan, called CS_RSOM_, in which $y$ (fast-scanning direction) and $x$ (slow-scanning direction) are the lateral coordinates defined by the scanning path and $z$ is the depth, which is perpendicular to the scanning plane and parallel to orientation of the ultrasound detector (see Fig. 1a). In this work a 3D slice refers to a cross-sectional volume with one voxel thickness perpendicular to one of the three axes ($x,y,z$) at a certain position.

### Frequency band equalization

RSOM’s capability of multi-scale imaging is exploited by FBE. FBE prevents small structures, which generate high frequency signals with lower intensity, from being obscured by the higher intensity signals of larger vessels. Therefore, RSOM sinograms are separated into two different frequency bands (LF: 10–40 MHz and HF: 40–99 MHz) using band-pass filters and reconstructed as two independent scans. Both the LFR and the HFR are then projected along the $x$-, $y$- or $z$-axis and merged into composite 2D images with the LFR and HFR marked in different color scales. The projections are done by applying the two visualization approaches, MIP or eMIP, that we aim to compare.

To ensure visual coherence and enhance feature visibility, the contrast of the color channels are matched to each other and optimized using techniques specific to the projection method and detailed under the following subheadings:

1. Alpha optimization for MIP,
2. Dynamic contrast optimization for eMIP.

### Alpha optimization for MIP

According to the FBE method, as published in ref. ^11^ (an advancement of refs. ^39–41^), the resulting 2D images ${{im}}_{{HF}}$ and ${{im}}_{{LF}}$ from projecting the LFR and HFR, respectively, along the desired axis, are equalized by weighting ${{im}}_{{HF}}$ with ${\alpha }^{* }$ as follows,

1 $${{im}}_{{HF}}^{* }={\alpha }^{* }{\cdot {im}}_{{HF}},$$

where ${\alpha }^{* }$ is determined by solving the following optimization problem,

2 $${\alpha }^{* }=\mathop{{\rm{argmin}}}\limits_{\alpha }{||}{{im}}_{{LF}}-\alpha \cdot {{im}}_{{HF}}{||}.$$

Then, ${{im}}_{{HF}}^{* }$ and $i{m}_{{LF}}$ are fused into an RGB image using linear scaling (range 0 to 1) between the joint minimum and maximum value, i.e.,

3 $$i{m}_{{green}}=\frac{i{m}_{{HF}}^{* }-i{m}_{\min }}{i{m}_{\max }-i{m}_{\min }}\;{\rm{and}}\;i{m}_{{red}}=\frac{i{m}_{{LF}}^{* }-i{m}_{\min }}{i{m}_{\max }-i{m}_{\min }}$$

with $i{m}_{\min }=\min ([i{m}_{{HF}}^{* },i{m}_{{LF}}])$ and $i{m}_{\max }=\max \left(\left[i{m}_{{HF}}^{* },i{m}_{{LF}}\right]\right)$, where $i{m}_{{green}}$ is assigned to the green channel and $i{m}_{{red}}$ to the red channel. Usually, for improved visibility, the contrast is adjusted by saturating lower and higher values according to an upper and lower threshold^39^ (in refs. ^11,12,19–22^ the saturation is not explicitly stated, but can be visually obtained). Since manual contrast adjustments are impractical for large datasets, and the objective is to achieve automated and standardized visualization, we used the default saturation values of 0.06 (lower contrast threshold) and 0.35 (upper contrast threshold) as set by our research facility.

### Dynamic contrast adjustment for eMIP

In order to enhance the representation of the skin microvasculature in the eMIPs, we implemented a dynamic contrast adjustment in our visualization method (eMIP). Unlike alpha optimization for MIP, the dynamic contrast adjustment saturates the images separately in the red and green color channel. For this reason, two upper thresholds $t{h}_{{HF}}^{+}$ and $t{h}_{{LF}}^{+}$ are determined for the two frequency-band-filtered reconstructions LFR and HFR, respectively. The upper thresholds are calculated by

4 $$t{h}_{{LF}}^{+}{=1.25\cdot q}_{0.95}\left(z{-{\text{MIP}}}_{{LF}}\right),$$

5 $$t{h}_{{HF}}^{+}{=1.25\cdot q}_{0.95}\left(z{-{\text{MIP}}}_{{HF}}\right),$$

where ${q}_{0.95}$ is the 95^th^ percentile function and $z{-{\text{MIP}}}_{{LF}}$ and $z{-{\text{MIP}}}_{{HF}}$ are the maximum intensity projections in $z$-direction of LF and HF, respectively. Percentiles are used instead of absolute maxima to avoid the influence of outliers caused by artifacts or isolated strong absorbers. Calculating the thresholds from the $z$-MIPs rather than the full 3D volumes increases robustness, as the $z$-MIPs are less affected by background noise and reconstruction sparsity. This is particularly important when the total reconstruction depth is large, where deeper regions contain many near-zero voxels due to signal attenuation and fluence decay. A detailed analysis of the robustness of the percentile choice is provided in Supplementary Note 2, Supplementary Fig. 2 and Supplementary Table 6.

Next, we apply eMIP along the chosen axis ($x$, $y$, or $z$) for LFR and HFR and term the received 2D images ${{im}}_{{HF}}\;{\rm{and}}\;i{m}_{{LF}}$, respectively. Subsequently, the images are zero clipped, capped by their corresponding upper threshold and linearly mapped to the range from 0 (zero values) to 1 (maximum values), i.e.,

6 $$i{m}_{{green}}=\frac{i{m}_{{HF}}^{* }}{t{h}_{{HF}}^{+}}\;{\rm{and}}\;i{m}_{{red}}=\frac{i{m}_{{LF}}^{* }}{t{h}_{{LF}}^{+}}$$

with $i{m}_{{HF}}^{* }=\max \left(\min \left(i{m}_{{HF}},{{th}}_{{HF}}^{+}\right),0\right)$ and $i{m}_{{LF}}^{* }=\max \left(\min \left(i{m}_{{LF}},t{h}_{{LF}}^{+}\right),0\right).$ Finally, the RGB image is composed by assigning $i{m}_{{red}}$ to the red channel and $i{m}_{{green}}$ to the green channel.

### Conventional maximum intensity projection (MIP)

We implemented MIP as the reference visualization method for rendering 3D slices into 2D images. This technique selects the highest intensity value of all 3D slices along each projection line through the reconstructed volume to create the 2D images. As there are three main orthogonal directions that can be defined by the RSOM coordinate system CS_RSOM_ when scanning, three 2D images can be rendered from the 3D volume; the $x$-MIP is produced by a projection along the $x$-axis (slow-scanning direction), the $y$-MIP by a projection along the $y$-axis (fast-scanning direction), and the $z$-MIP by a projection along the $z$-axis (depth).

### Enhanced maximum intensity projection (eMIP)

Our eMIP method includes a skin surface alignment algorithm that shifts $z$-columns (lines of single voxels at a fixed ${xy}$ position stacked along the $z$-axis in the 3D volumetric array) of the volumetric reconstruction in order to project the skin boundary into a 3D slice parallel to the lateral directions at a predefined depth with respect to the CS_RSOM_.

The skin surface is aligned using two sub-algorithms: surface detection and surface correction algorithms. The surface detection algorithm determines a surface function that approximates the skin surface within the HFR and LFR. Then, the surface correction algorithm transforms LFR and HFR according to the surface function so that the surface is at a uniform level within.

Once the surface correction is completed, maximum intensities are projected analogously to the MIP process. The projections along the $x,y$ and $z$ axes are termed $x$-eMIP, $y$-eMIP and $z$-eMIP, respectively.

### Surface correction

We describe the LFR and HFR by their voxel intensity values ${V}_{{ijk}}$, with $\left(i,j,k\right)\in {{D}_{{xyz}}}:= D_{x}\times {D}_{y}\times {D}_{z}:= \left\{1,\ldots ,{n}_{x}\right\}\times \left\{1,\ldots ,{n}_{y}\right\}\times \left\{1,\ldots ,{n}_{z}\right\}$ representing the spatial position of the voxel within the volume, where ${n}_{x}$, ${n}_{y}$ and ${n}_{z}$ are number of voxels along the respective dimension. Furthermore, we name the vector $[{V}_{{ij}1},\ldots ,{V}_{{ij}{n}_{z}}]$ the $z$-column $(i,j)$.

The surface correction algorithm first evaluates the surface function $(x,y)\mapsto {sfit}\left(x,y\right)$ from the surface detection algorithm at each point $\left(i,j\right)\in {{D}_{{xy}}}:= D_{x}\times {D}_{y}$ and projects the results to the nearest value in ${D}_{z}$, i.e.,

7 $${s}_{{ij}}=\mathop{{\rm{argmin}}}\limits_{k\in {D}_{z}}{|sfit}(i,j)-{k|}.$$

Then, each $z$-column in LFR and HFR is shifted, so that the entries ${V}_{{ij}{s}_{{ij}}}$ are aligned at the same level, called the zero-level. The shifted reconstructions are assembled such that they preserve their size, and the zero level is placed at *k* = 100 (equivalent to a depth of 400 µm). Values which are shifted out of the volume limits are cut off, and missing values are padded with zeros.

### Surface detection

The surface detection algorithm utilizes the least squares approach to estimate the best fitting polynomial to describe the skin surface. Typically, the skin surface is tilted with respect to the 3D volume, and in some cases curved due to heterogeneous pressure from the attached scanning device. A tilt (rotation of the surface normal against the $z$-driection) can be expressed by a simple linear function and our experience suggests that the curvature of the surface is well approximated by a 2D polynomial of degree two or three. Moreover, interpolating or fitting high-degree polynomials can be prone to numerical instability and the Runge phenomenon, especially if not approached with care^42,43^. Consequently, we limited the polynomial degree to a maximum of 4.

The iterative surface detection process comprised of four general building blocks (simplified flowchart in Fig. 1 and detailed flowchart in Supplementary Fig. 3). Given the 3D reconstructed volumes LFR and HFR, the algorithm identifies the first voxels in the $z$-direction that exceed a certain intensity level (referred to as surface points) and then fits a polynomial function that accurately approximates the depth of the surface points given their $x$ and $y$ position. The former subroutine forms the “Find surface points” block, and the latter, the “Fit Surface Function” block. To avoid misidentification of the signal of hairs, artifacts, noise, or other high absorbing structures (representatively visualized in Fig. 1g, h) as surface points, we added two more sub-routines (blocks) that remove the influence of artifacts (Fig. 1i, j) and ensure uniform and smooth representation of the skin surface (“Exclude Outliers” and “Calculate Feasible Range”, respectively). By means of outer and inner iterative loops, we excluded such outliers, narrowed the search range, selected more suitable surface points and updated the polynomial function until we obtained a final surface function.

The building blocks of our skin surface detection process required the tuning of hyperparameters as explained below. The hyperparameters used are shown in Supplementary Tables 2–5 and these were manually optimized over several iterations on a subset of 139 images, of which 69 were randomly selected and 70 were handpicked based on complexity to represent a broad range of possible cases (e.g., scans with noise, reflections, hairs or weak signal). The five building blocks are:

1. Find Surface Points,
2. Fit Surface Function,
3. Outlier Exclusion,
4. Calculate Feasible Range,
5. Final Surface Function.

This block “Find Surface Points” searches for points on the skin surface. A surface point ${u}_{i}=\left({x}_{i},{y}_{i},{z}_{i}\right)$ is given by the coordinates of a voxel ${V}_{{x}_{i}{y}_{i}{z}_{i}}$ (see Fig. 1j, k). The surface points, in terms of $z$-column coordinates, are selected separately for LFR and HFR. The union of all surface points (from LFR and HFR) constitutes set $S$.

In each $z$-column $\left({x}_{i},{y}_{i}\right)$ of LFR and HFR, we select at most one surface point ${u}_{i}=\left({x}_{i},{y}_{i},{z}_{i}\right),$ for which the entry ${V}_{{x}_{i}{y}_{i}z}$ is greater or equal than a threshold $t{h}_{t}$, such that

8 $${z}_{i}=\inf \{{z\in \rho ({x}_{i},{y}_{i}):V}_{{x}_{i}{y}_{i}z}\ge t{h}_{t},\left({x}_{i},{y}_{i}\right)\in {D}_{{xy|t}}\},$$

where $\rho$ is the search range (see building block 4). If no entry fulfils the criteria $({z}_{i}=\infty )$, the surface point ${u}_{i}$ will be omitted.

The thresholds $t{h}_{t}$ are derived locally on a raster of ${D}_{{xy}}$ (see Fig. 1g) to ensure that a sufficient number of points are selected in low-intensity voxel regions while reducing noise and artifact detection in high-intensity areas. Additionally, the approach of local thresholds guarantees an adequate number of surface points near edges and corners, which is crucial for accurately fitting the surface polynomial. The sub-domains ${D}_{{xy|t}}$ of the raster (referred to as raster tiles) are defined for $t=1,\ldots ,16$ by

9 $${D}_{{xy}|t}=\left\{\left({x}_{i},{y}_{i}\right)\in {D}_{{xy}}:{a}_{{\left[t-1\right]}_{4}+1} < {x}_{i}\le {a}_{{\left[t-1\right]}_{4}+2},{b}_{{\lceil}t/4{\rceil}} < {y}_{i}\le {b}_{{\lceil}t/4{\rceil}+1}\right\}$$

with ${a}_{j}=\left\lfloor {d}_{j}{n}_{x}+1/2\right\rfloor$, ${b}_{j}=\left\lfloor {d}_{j}{n}_{y}+1/2\right\rfloor$ for $d=\left[0,\,0.15,\,0.5,\,0.85,\,1\right]$ and ${\left[t\right]}_{4}:=t\,(\mathrm{mod}\,4)$. The threshold $t{h}_{t}$ is calculated on the respective raster tile ${D}_{{xy|t}},$ with

10 $$t{h}_{t}=\max \left\{{\frac{{\rm{\tau }}}{\text{sensitivity}}}q_{\alpha }(z{-{\text{MIP}}}_{\rho ,t}),{\frac{0.15\cdot {\rm{\tau }}}{\text{sensitivity}}}q_{{\alpha }_{{relaxed}}}(z{-{\text{MIP}}}_{\rho })\right\},$$

where ${q}_{\rm{\alpha }}(\cdot)$ is the ${\rm{\alpha }}$-quantile function, ${z\text{-MIP}}_{\rho }$ the maximum intensity projection along the $z$-direction restricted to the surface range $\rho ,$ and $z{-{\text{MIP}}}_{\rho ,t}$ is additionally restricted to tile ${D}_{{xy|t}}$. The second term of the maximum imposes a (global) lower limit, reducing the detection of pure noise in areas with no or very weak signal. The relaxation values 0.15 and ${\alpha }_{{relaxed}}=\alpha -\frac{1}{3}\left(1-\alpha \right)$ were chosen empirically, where slightly higher or lower values have been demonstrated to produce similar results.

Multiplier $\frac{{\rm{\tau }}}{\text{sensitivity}}$ and quantile $\alpha$ are fine-tuned according to Supplementary Tables 2 and 3, where in the 4^th^ iteration quantile $\alpha$ is determined adaptively according to the noise level immediately above the estimated surface. First, we select the noise range of 41 voxels (empirically shown to be robust) around the top boundary of the surface range *ρ*, i.e.,

11 $${\rho }_{{noise}}=[{\rho }_{{top}}-20,{\rho }_{{top}}+20].$$

Then, we calculate the target threshold $t{h}_{{target}}$ by:

12 $$t{h}_{{noise}}={q}_{0.95}\left(z{-{\text{MI}}}{\text{P}}_{{noise}}\right),$$

13 $$t{h}_{\min /\max }={\frac{1}{\text{sensitivity}}q}_{{\alpha }_{\min /\max }}\left(z\text{-MIP}\right),$$

14 $$t{h}_{{target}}=\min\, (\max (t{h}_{{noise}},t{h}_{\min }),t{h}_{\max }),$$

where $z{-{\text{MIP}}}_{noise}$ is the $z$-MIP restricted to the noise range ${\rho }_{{noise}}$. The adapted alpha ${\alpha }_{{adpt}}$ is chosen such that the following two equations are fulfilled:

15 $$\left\{\begin{array}{c}t{h}_{{target}}=\frac{1}{\text{sensitivity}}{q}_{{\alpha }_{{relaxed}}}(z\text{-MIP}),\\ {\alpha }_{{relaxed}}={\alpha }_{{adpt}}-\frac{1}{3}\left(1-{\alpha }_{{adpt}}\right).\end{array}\right.$$

With the adaptive alpha, the algorithm finds more surface points in the selection process which are closer to the actual surface without falsely taking noise into account.

The second building block “Fit Surface Function” fits a function ${sfit}$ to the surface points $S$ or, if available, ${S}_{{inc}}$ (defined in building block 3). The function ${sfit}$ referred to as surface function is modeled as a polynomial of degree $\left(n,m\right)$, i.e.,

16 $${sfit}\left(x,{y;n},m\right)=\sum _{(i,k)\in I}{{c}_{{ik}}^{* }x}^{i}{y}^{k},$$

with $I=\{\left(i,k\right)\in {{\mathbb{N}}}^{2}:i\le n,k\le m,i+k\le \max (n,m)\}$. In Fig. 1j, k we illustrate the process. The coefficients ${c}^{* }={\left[{c}_{00}^{* },{c}_{10}^{* },\ldots {,c}_{0m}^{* }\right]}^{T}$ are either determined by solving the linear least square problem (called “Polyfit”), or the RANSAC algorithm^44^, which is known to be robust against outliers. At the first few iterations a linear (degree (1,1)) polynomial model is chosen to robustly obtain a rough estimate of the surface location. In later iterations, the polynomial degree is selected dynamically.

In the Polyfit algorithm, the coefficients ${c}_{{nm}}$ for varying polynomial degrees $(n,m)$ are determined on a training set (95% of ${S}_{{inc}}$) by:

17 $${c}_{{nm}}=\mathop{{\rm{argmin}}}\limits_{c\in {{\mathbb{R}}}^{{|I|}}}{\left|\left|\left[\begin{array}{cccc}1 & {x}_{s}^{1}{y}_{s}^{0} & \cdots & {x}_{1}^{0}{y}_{1}^{m}\\ \vdots & \vdots & \ddots & \vdots \\ 1 & {x}_{s}^{1}{y}_{s}^{0} & \cdots & {x}_{s}^{0}{y}_{s}^{m}\end{array}\right]c-\left[\begin{array}{c}{z}_{1}\\ \vdots \\ {z}_{s}\end{array}\right]\right|\right|}_{2}.$$

The polynomial degree $\left({n}^{* },{m}^{* }\right)$ with minimal cost will be selected. The cost is evaluated on the test set ${S}_{{test}}$ by:

18 $$cos{t}_{{nm}}=p(n,m)\cdot \sum _{{u}_{i}\in {S}_{{test}}}{\left({sfit}\left({x}_{i},{y}_{i}{;n},m\right)-{z}_{i}\right)}^{2}$$

with a penalty factor $p\left(n,m\right)={1.01}^{{\left(m-1\right)}^{2}+{\left(n-1\right)}^{2}}$ on the polynomial degree $(n,m)$. The penalty is introduced to favor low polynomial degrees, because they are more robust (e.g., Runge phenomenon). The coefficients ${c}^{* }$ of the final function ${sfit}$ with polynomial degree $\left({n}^{* },{m}^{* }\right)$ are obtained by fitting the entire set ${S}_{{inc}}$ once more.

The RANSAC algorithm is only used to fit linear functions using a sample size of 10 and a maximum distance of 30 for inlier points (for details see ref. ^44^).

The order of application and hyperparameter are clarified in Supplementary Table 5.

The block “Outlier Exclusion” assembles a subset of surface points ${S}_{{inc}}\subseteq S$ which exclude outliers (illustrated by blue and red points in Fig. 1i, j, respectively). Here, we define outliers to be surface points which exceed a certain distance from ${sfit}$ with the exact definition given in the following. For notational purposes, we identify the previous set ${S}_{{inc}}$ (or $S$ after an iteration of the “Find Surface Points” building block) as ${S}_{{inc}}^{{old}}$. First, we calculate the residuals ${r}_{j}$ for ${u}_{j}=\left({x}_{j},{y}_{j},{z}_{j}\right)\in {S}_{{inc}}^{{old}},$ and assemble these residuals into the vector $r=\left[{r}_{1},\ldots ,{r}_{s}\right],$ whereby

19 $${r}_{j}={sfit}\left({x}_{j},{y}_{j}\right)-{z}_{j}.$$

Then we derive the standard deviation $\sigma ={std}\left(r\right),$ and the local second moments ${\gamma }_{t}^{2}=E\left[{r}_{t}^{2}\right],$ where ${r}_{t}=[{r}_{{t}_{1}},\ldots ,{r}_{{t}_{s}}]$ is assembled by all residuals ${{r}_{t}}_{i}$ corresponding to raster tile $t$, i.e., $\left({x}_{{t}_{i}},{y}_{{t}_{i}}\right)\in {D}_{{xy|t}}$. Globally, we define $\gamma (x,y)$ by ${\gamma }_{|\left(x,y\right)\in {D}_{{xy|t}}}={|\gamma }_{t}|$. Introducing *γ* ensures enough surface points in ${S}_{{inc}}$ in each raster tile. Next, we introduce the thresholds

20 $${h}_{{top}}(x,y)=\min \left(\max \left({\sigma }_{{top}}\cdot \sigma ,{\gamma }_{{top}}\cdot \gamma (x,y)\right),{h}_{{abs}}\right),$$

21 $${h}_{{bot}}(x,y)=\max \left({\sigma }_{{bot}}\cdot \sigma ,{\gamma }_{{bot}}\cdot \gamma (x,y)\right),$$

where the distance ${h}_{{abs}}$ is introduced to consistently remove reflections. The hyperparameter ${h}_{{abs}}$, ${\sigma }_{{top}}$, ${\sigma }_{{bot}},$ ${\gamma }_{{top}}$ and ${\gamma }_{{bot}}$ are defined for each iteration in Supplementary Table 5. Surface points which are ${h}_{{top}}$ above or ${h}_{{bot}}$ below ${sfit}$ (loosely speaking a factor of *σ* or *γ*) will be excluded. Hence,

22 $${S}_{{inc}}=\left\{{u}_{i}=\left({x}_{i},{y}_{i},{z}_{i}\right)\in S:-{h}_{{bot}}\left({x}_{i},{y}_{i}\right) < {sfit}\left({x}_{i},{y}_{i}\right)-{z}_{j} < {h}_{{top}}\left({x}_{i},{y}_{i}\right)\right\}.$$

The fourth building block “Calculate Feasible Range” ensures that the surface points for each $z$-column are inside a range defined by the surface function derived in the previous iteration (or outer loop). The surface range $\rho \left(x,y\right)=[{\rho }_{{bot}}\left(x,y\right),{\rho }_{{top}}\left(x,y\right)]$ defines the interval along the $z$-column $(x,y)$ within which surface points can be selected. The limits ${\rho }_{{bot}}\left(x,y\right)$ and${\rho }_{{top}}\left(x,y\right)$ are determined by

23 $${\rho }_{{bot}}\left(x,y\right)={sfit}\left(x,y\right)-{\theta }_{{bot}}{\text{and}}\,{\rho }_{\rm{top}}={sfit}\left(x,y\right)+{\theta }_{\rm{top}},$$

where ${\theta }_{{bot}}$ and ${\theta }_{{top}}$ are defined in Supplementary Table 2.

In the last building block “Final Surface Function” a small offset is added to the function ${sfit}$, so that the estimated surface is above most surface points. The offset is calculated by

24 $${\rm{offset}}=r+\frac{1}{5}s+3,$$

where $r$ is the 80th percentile and s is the standard deviation of the residuals (see Eq. (19)). The offset *r* ensures that the estimated surface is on top of at least 80% of the detected surface points and the remaining 20% are estimated by the standard deviation *s* plus an additional fixed offset of 3 voxels to eliminate confounding effects from possible outliers.

### Computational cost analysis

For the computational cost analysis, we visualized all 1725 scans using both eMIP and MIP in MATLAB 2025a on a MacBook Pro equipped with an Apple M4 Pro chip featuring a 20-core CPU and 48 GB of unified memory. We recorded the loading time, the time required to run the surface detection algorithm, the time needed for visualization (for eMIP without surface detection but with surface correction), and the time to save the resulting images to disk.

Reconstructions were performed on a high-performance workstation running Windows 10 Pro, equipped with an AMD Ryzen Threadripper 3970 × 32-core processor, 256 GB of RAM, and an NVIDIA GeForce RTX 3090 GPU. The system is built on a Gigabyte TRX40 DESIGNARE motherboard and is optimized for compute-intensive, parallel processing tasks. The reconstructions algorithm^6^ was executed in MATLAB 2024b using precomputed sensitivity fields and CUDA-based GPU implementation for acceleration. We recorded the total reconstruction time for all scans, including raw data loading from SSD, reconstruction of HFR and LFR volumes, and saving to disk.

For all recorded durations, we computed the mean, median, standard deviation, and maximum values.

### Perceptual image quality assessment by experts

The quantification in terms of perceived image quality is the metric employed in this work to validate eMIP against the conventional visualization method for RSOM images. Perceptual (or subjective) IQA is based on the opinion of qualified observers to evaluate the appearance of an image and its suitability for a specific purpose and it is considered in literature the gold standard method, due to its reliability^45,46^. Eight optoacoustic imaging experts quantified the visual quality of 1725 RSOM reconstructed scans, with each scan rated at least twice, once after visualization with MIP and the conventional contrast from FBE and once after visualization with eMIP using FBE with our dynamic contrast adjustment. The raters were blinded to the visualization method and the images were presented individually in an independently randomized order for each rater using a unique seed, preventing any systematic bias during the scoring process. To facilitate quality evaluation by the experts, we implemented a graphical user interface (GUI) (see Supplementary Fig. 4), that shows the MIPs along the three dimensions of the reconstructed volumes for each of the scans. The experts were then asked to rate the visualization in one of four categories, bad quality (1), medium quality (2), good quality (3) and perfect quality (4), according to their opinion of the usability of the data in posterior data analysis. It was possible to postpone the evaluation of an image for later evaluation or to exclude an image completely. The scans were randomly shuffled for each participant, including 100 repeated scans for each visualization method for quantification of intra-expert consistency. The final image quality rating was calculated by averaging the scores of all eight raters (MOS). When a scan received two different ratings from the same rater (for the 100 repeated scans), the rater’s score for the scan was taken to be the average of the two scores (before calculating the MOS).

### Perceived quality distribution & significance of MOS differences between MIP and eMIP

The distribution of the MOS is presented by a histogram (Fig. 2g). The MOS values range from 1 (poor quality) to 4 (excellent quality). The scores are grouped into bins with a width of 0.125, and the height of each bin represents the percentage of scans within that range. MOS values that fall on a bin boundary are included in the bin to the left.

To evaluate the statistical significance of the differences in perceived image quality, we performed two tests:

1. A two-sided two-sample t-test to assess whether the mean MOS for eMIP was significantly greater than the mean MOS for MIP. However, because of discrete data points, the assumption of normality may not be valid.
2. A two-sided Wilcoxon signed-rank test to determine the significance that the median MOS of eMIP is greater than the median MOS of MIP.

For both tests we set the significance level to *α* = 0.01%. Additionally, we reported the confidence interval (CI) for the mean MOS differences corresponding to this significance level. To derive the CI for the median differences to the significance level *α* we used bootstrapping.

### Comparison of pairs of images generated by MIP and eMIP

To compare eMIP and MIP images rendered from the same RSOM scan we calculated the differences *d*_*i*_ between the MOS values for all 1725 pairs of images ($i{m}_{i,{eMIP}}\;{\&}\;i{m}_{i,{MIP}}$) as follows:

25 $${d}_{i}={MOS}\left({sca}{n}_{i,{eMIP}}\right)-{MOS}\left({sca}{n}_{i,{MIP}}\right).$$

Then, we represented the distribution of all differences ${d}_{i}$ in a histogram (Fig. 2i) centered at zero with a bin width of 1/3. A substantial difference was defined as $|{d}_{i}|\ge 0.5$.

### Inter- and intra-expert agreement

The inter-expert agreement, the degree to which different experts agree with each other when rating the same image, and intra-expert agreement, the degree to which the same expert rates the same image at different times, for both MIP and eMIP was quantified by calculating the generalized weighted Fleiss’ kappa coefficient (${\hat{\kappa }}_{{inter;MIP}}$ ${\hat{\kappa }}_{{inter;eMIP}}$ and ${\hat{\kappa }}_{{intra;MIP}}$, ${\hat{\kappa }}_{{intra;eMIP}}$)^47–51^ for multiple raters. A linear weighting matrix was used, with weights

26 $${w}_{{ij}}=\frac{{|i}-{j|}}{3},$$

where $i,j$ are the ratings from 1 to 4. To quantify intra-expert agreement (${\hat{\kappa }}_{{intra}}$), the 100 scans that were assessed twice by each rater were used to calculate the weighted Fleiss’ kappa coefficients, and the mean value was reported. The inter- and intra-expert agreement gain are defined by

27 $${\hat{\kappa }}_{{inter;gain}}={\hat{\kappa }}_{{inter;eMIP}}-{\hat{\kappa }}_{{inter;MIP}},$$

28 $${\hat{\kappa }}_{{intra;gain}}={\hat{\kappa }}_{{intra;eMIP}}-{\hat{\kappa }}_{{intra;MIP}}.$$

By definition, *κ* ranges from −1 and 1, where *κ* = 0 is the agreement expected by chance. A *κ* value between 0 and 1 represents levels of agreement from slight to perfect (*κ* = 1). While there is some debate on how to interpret values between 0 and 1, we present the benchmark proposed in ref. ^52^ in Table 1.

The standard errors (*SE*) of the agreement coefficients $\hat{\kappa }$ were derived from the square root of the unconditional variance *V*, which is the sum of the variances ${V}_{{im}}$ (random selection of images) and ${V}_{r}$ (sampling of raters), i.e., $V={V}_{r}+{V}_{{im}}$^49^. While there exists a closed formula to estimate ${V}_{{im}}$^49,50^, ${V}_{r}$ was estimated by the delete-one Jackknife variance estimator (inter-expert agreement) and the usual unbiased variance estimator divided by 8 (intra-expert agreement).

The variance of the inter-expert agreement gain ${\hat{\kappa }}_{{inter;gain}}=$ ${\hat{\kappa }}_{{inter;eMIP}}-{\hat{\kappa }}_{{inter;MIP}}$ was estimated by

29 $${v}_{{inter;gain}}={v}_{{r;inter;gain}}+{v}_{{im;inter;MIP}}+{v}_{{im;inter;eMIP}},$$

where ${v}_{{r;inter;gain}}$ was derived by the delete-one Jackknife variance estimator. Furthermore,

30 $${v}_{{intra;gain}}={v}_{{r;intra;gain}}+{v}_{{im;intra;MIP}}+{v}_{{im;intra;eMIP}},$$

and ${v}_{{r;intra;gain}}$ was estimated by variance estimator for the paired kappa differences of each rater (usual unbiased variance estimator).

The limits of the 95% CI are derived by

31 $${L}_{{upper}/{lower}}=\hat{\kappa }\pm (1.96\cdot {SE}).$$

We analyzed the agreement rate of the MOS of groups of 3 raters. For this we created all possible combinations of groups of three raters and calculated their MOS for each scan. Then we calculated the weighted Fleiss’ kappa between all pairs of two groups with distinct elements (i.e., all different raters) and reported the mean kappa ${\hat{\kappa }}_{g3}$. The unconditional variance is estimated by

32 $${v}_{g3}={v}_{{r;g}3}+{v}_{{im;g}3},$$

where ${v}_{{im;g}3}=\mathop{\sum }\limits_{i}^{P}{v}_{{im;g}3{;i}}$ with ${v}_{{im;g}3{;i}}$ being the variance estimators for the random selection of the images for all valid group pairs $$i=1,\ldots ,P=\left(\begin{array}{c}8\\ 2\end{array}\right)\left(\begin{array}{c}6\\ 3\end{array}\right)=560$$. The variance ${v}_{{r;g}3}$ was estimated by the delete-two Jackknife variance estimator^53^. For this all $$Q=\left(\begin{array}{c}8\\ 2\end{array}\right)$$ combinations of six experts were built and for each of these sets the mean kappa ${\hat{\kappa }}_{l}$ of all $$\left(\begin{array}{c}6\\ 3\end{array}\right)$$ pairs of two groups with distinct elements was calculated. The variance estimation of ${v}_{{r;g}3}$ is then derived by

33 $${v}_{{r;g}3}=\frac{8-2}{2\cdot 8}\mathop{\sum }\limits_{l=1}^{Q}{\left({\hat{\kappa }}_{l}-{\hat{\kappa }}_{g3}\right)}^{2}.$$

The variance of the agreement gain ${\hat{\kappa }}_{g3{;gain}}={\hat{\kappa }}_{g3{;eMIP}}-{\hat{\kappa }}_{g3{;MIP}}$ was estimated by

34 $${v}_{g3{;gain}}={v}_{{r;}{\rm{g}}3{;gain}}+{v}_{{im;}{\rm{g}}3{;MIP}}+{v}_{{im;}{\rm{g}}3{;eMIP}},$$

where ${v}_{{r;inter;gain}}$ was derived by the delete-two Jackknife variance estimator.

The hypothesis tests shown in Fig. 2i are defined as follows with significance level *α* = 5%:

- Test 1 (${\hat{\kappa }}_{g3} > {\hat{\kappa }}_{{inter}}$): The *p*-value of ${\hat{\kappa }}_{{inter;gain}} > 0$ is determined by
  35 $$p=1-\Phi \left(\frac{{\hat{\kappa }}_{g3}-{\hat{\kappa }}_{{inter}}}{\sqrt{{v}_{g3}+{v}_{{inter}}}}\right),$$
  where Φ is the cumulative distribution function of the normal distribution.
- Test 2 (${\hat{\kappa }}_{{inter;gain}} > 0$): The *p*-value of ${\hat{\kappa }}_{{inter;gain}} > 0$ is determined by
  36 $$p=1-\Phi \left(\frac{{\hat{\kappa }}_{{inter;gain}}}{\sqrt{{v}_{{inter;gain}}}}\right).$$
- Test 3 (${\hat{\kappa }}_{{intra;gain}} > 0$): analogous to Test 2.
- Test 4 (${\hat{\kappa }}_{g3{;gain}} > 0$): analogous to Test 2.

### Inclusion & ethics statement

All authors included in this work have fulfilled the criteria for authorship required by Nature Portfolio journals, and their contributions were essential for the development of algorithms, the data acquisition, and the analysis and interpretation of the results. This research includes findings that are locally relevant and have been determined in collaboration with local partners. Additionally, local and regional research relevant to this study was considered in citations. This research has not been severely restricted or prohibited in the setting of the researchers and does not result in stigmatization, incrimination, discrimination or personal risk to participants.

The data used for validating the algorithm was acquired as part of three clinical studies in two institutions: University Hospital Klinikum rechts der Isar from Technical University of Munich (Germany) and Tübingen University Hospital (Germany). The three clinical studies conformed to the ethical principles for medical research involving human participants in accordance with the Declaration of Helsinki. All participants provided written informed consent.

Ethical approval for the two clinical studies in the University Hospital Klinikum rechts der Isar from Technical University of Munich were obtained from Ethics Committees in the Technical University Munich with the following protocol IDs: Diabetes study: 109/17 S and CVD study: 326/19 S.

The clinical study conducted by Klinikum University Tübingen was the Prediabetes Lifestyle Interventional Study^54^ (PLIS), registered under ClinicalTrials.gov with ID NCT01947595, and a the Ethics Committee at the University Hospital of Tübingen approved the study protocol.

## Supplementary information

Supplementary Information

## Data Availability

This work aimed to develop an image rendering technique (eMIP). For this purpose, we only employed the acquisitions using non-invasive raster-scan optoacoustic mesoscopy (RSOM Explorer C50®, iThera Medical GmbH) and we did not use clinical or socio-demographic data from the clinical studies. Since the patient dataset was collected as part of specific clinical studies (approval numbers provided in this manuscript), the data with annotated clinical information cannot be made publicly available due to data protection regulations. The data that support the findings and were generated as part of this manuscript are available from the corresponding author on reasonable request.

## Code availability

The underlying code for this study is available on GitHub and can be accessed via this link https://github.com/juestellab/eMIP.
