# Supplementary material for: Enhanced maximum intensity projection (eMIP) for improving the fidelity of optoacoustic images
Source: Npj Imaging. 2025 Oct 6;3:49. doi: 10.1038/s44303-025-00112-z (PMC12501383; doi:10.1038/s44303-025-00112-z)
Supplement: Supplementary file 1 — Supplementary Information [file 44303_2025_112_MOESM1_ESM.pdf]

Supplementary Information

Supplementary Notes

Supplementary Note 1. Consistency of eMIP's MOS gain across demographic and technical subgroups

To investigate the influence of different confounding factors with respect to differences in perceived image quality between eMIP and MIP within the data set, we conducted an analysis on different subgroups of the data – analogous to the analysis described in the Methods section of the main manuscript. This approach allowed us to evaluate the statistical significance of the differences in perceived image quality across the groups defined by the following possible confounders:

1. RSOM system (for each of the two systems corresponding to each clinical study)
2. Scan body location (leg/ arm)
3. Skin pigmentation (defined by the Fitzpatrick parameters I/II (light) and III/IV (darker))
4. Gender (female/ male)
5. BMI
6. Age
7. Low (MOS<1.5), medium (1.5≤MOS<2.5) or high (2.5≤MOS) baseline quality
8. Brightness of the z-MIP (i.e., mean pixel intensity of the grayscale image of the z-MIP):
   Scans were categorized based on the relative brightness between the MIP and eMIP visualizations as follows:
    a. Same brightness: Scans where the mean pixel intensity differs no more than 10%
    b. MIP brighter: Scans where the MIP z-MIP image is brighter
    c. eMIP brighter: Scans where the eMIP z-MIP image is brighter

Supplementary Table 1 collects the mean and median baseline MOS and the corresponding gain by eMIP (i.e., quality improvement) for the different groups of confounders. Additionally, we reported the CI for mean and median values with a significance level of α=0.01%.

Supplementary Note 2. Percentile based threshold selection for dynamic contrast

The two upper thresholds in the dynamic contrast adjustment — defined in Equation (4) and (5) in the main manuscript — are $th_{HF}^{+} = 1.25 \cdot q_{0.95}(z\text{-}MIP_{HF})$ and $th_{LF}^{+} = 1.25 \cdot q_{0.95}(z\text{-}MIP_{LF})$, where $q_{0.95}$ returns the 95th percentile of the respective 2D z-MIP based on the 95th percentile of the 2D z-MIP.

The following analysis demonstrates that a percentile-based thresholding approach offers greater robustness than relying on the absolute maximum value, which is often sensitive to noise or isolated outliers. In particular, we show that selecting any percentile between approximately the 80th and 97th percentile results in similarly consistent and reliable contrast adjustments. These findings support the use of the 95th percentile as a stable and empirically effective choice for thresholding in dynamic contrast enhancement.

On a random subset of 200 scans, we extracted percentile values from each z-MIP (projected separately from the LFR and HFR) at percentile ranks with a step size of 0.2%. These values were then normalized by the respective 95th percentile of each scan and reconstruction type. We subsequently computed for each rank and reconstruction type the mean and standard deviation across all scans.

This analysis is visualized in Supplementary Figure 2, which shows the normalized percentile curves for both HFR and LFR across 200 scans. The continuous curve shows the mean across all scans and the shaded region represents one standard deviation, highlighting the variability of percentile values across scans.

Notably, the curves remain relatively stable between the 80th and 97th percentiles with standard deviations below 0.1 for both reconstruction types. This confirms that the 95th percentile provides a reliable and robust threshold for dynamic contrast adjustment. In contrast, percentile ranks above the

98th show a sharp increase in variability (particularly in HFR) indicating growing sensitivity to noise and outliers. Vertical lines in the plots illustrate how different high-percentile ranks compare. The corresponding numerical values are summarized in Supplementary Table 6, further reinforcing the empirical validity of using the 95th percentile as a stable and generalizable choice.

Supplementary Figures

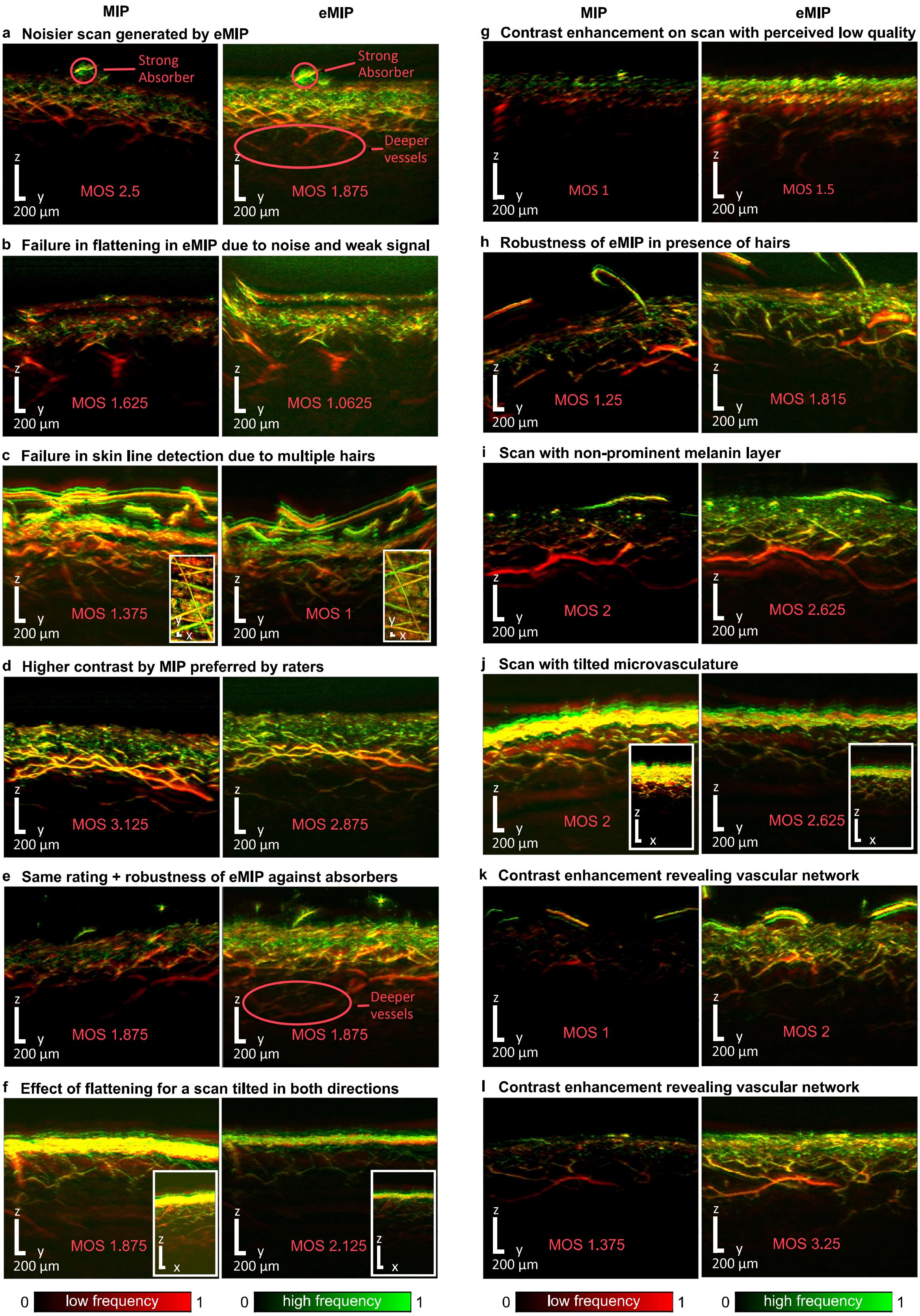

***Supplementary Figure 1. Comparison of representative clinical raster scan optoacoustic mesoscopy (RSOM) images from the dataset visualized by two methods:** For each case (**a-l**), a short title describes the main details of the scan. The mean opinion score (MOS) is shown in red for each visualization. MOS scores range from 1 (=bad) to 4 = (perfect).*

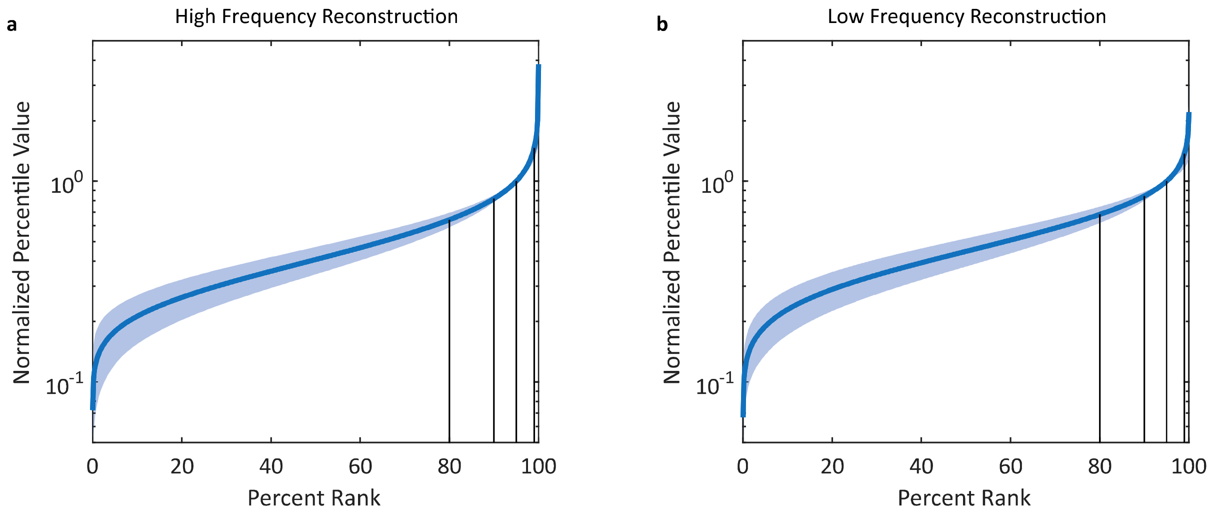

Supplementary Figure 2. Percentile curve analysis for dynamic contrast thresholding. *Mean normalized percentile values across 200 scans for the (**a**) high-frequency reconstruction (HFR) and (**b**) low-frequency reconstruction (LFR). Percentile values were normalized by the 95th percentile of each scan. The dark blue line shows the mean normalized value and shaded areas indicate ± one standard deviation across scans. Vertical lines denote selected percentiles (80th, 90th, 95th, 99th), illustrating the stability of values in the upper percentile range and the robustness of the 95th percentile choice.*

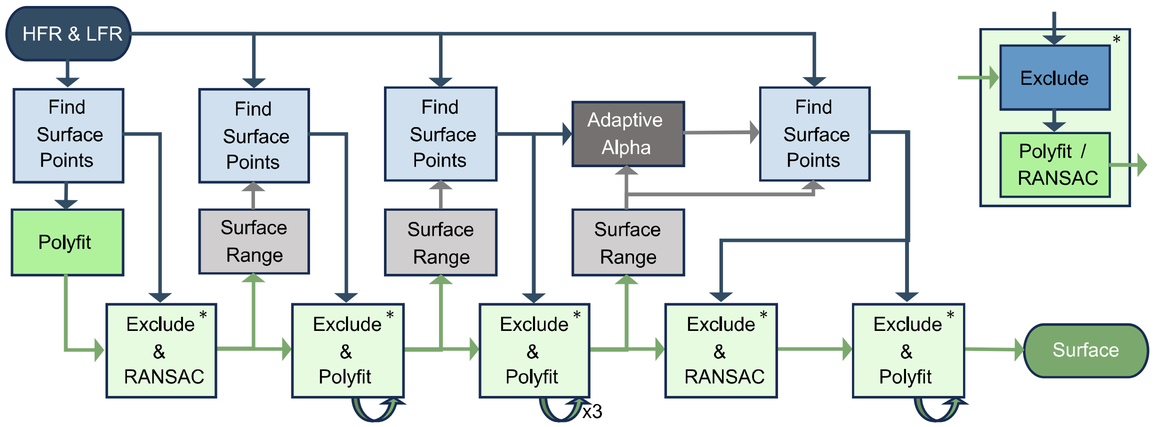

Supplementary Figure 3. Detailed surface detection workflow. *Inputs: low frequency reconstruction (LFR) and high frequency reconstruction (HFR) RSOM volumes. Output: surface function (polynomial). The workflow consists of the four building blocks: "Find Surface Points", "Calculate Feasible Surface Range" (short: "Surface Range"), "Fit Surface Function" (specified as "Polyfit" or "RANSAC" and dependent on the underlying fitting algorithm) and "Exclude Outliers" (short: "Exclude"). Additionally, the calculation of the adaptive alpha was added to the flowchart. The subroutine of the combined box "Exclude & Polyfit/RANSAC" is shown at the top right. The output of the building blocks is passed on according to the flowlines. U-arrows indicate repetition (in one case, possibly up to 3 times) of a block passing output to itself and overwriting matching input.*

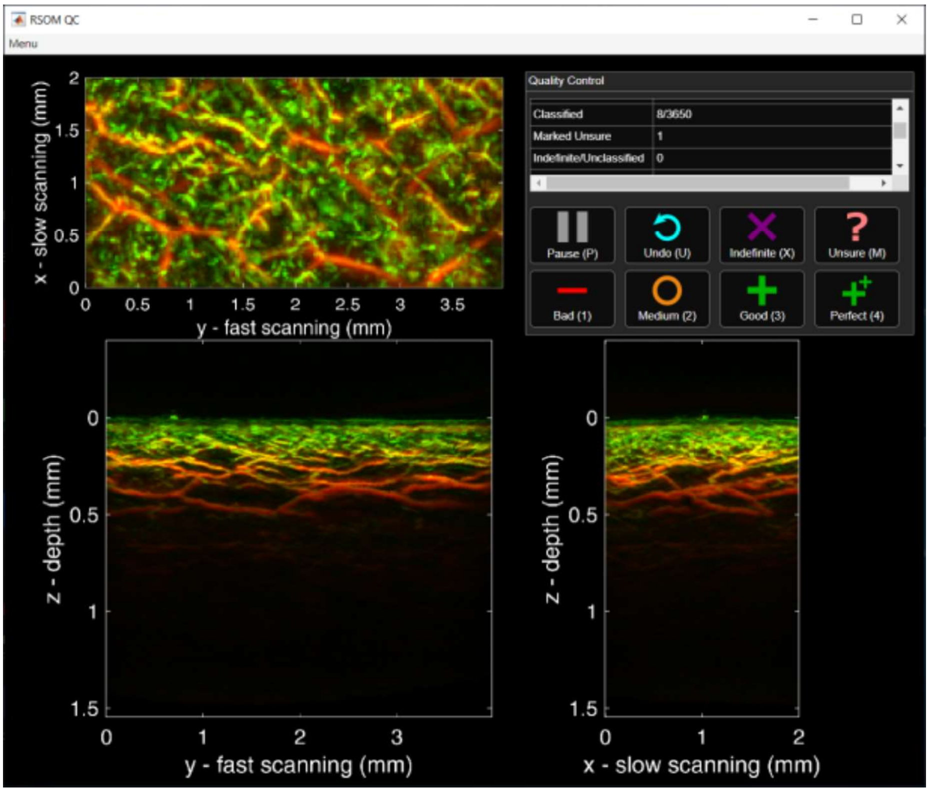

Supplementary Figure 4. Screenshot of the graphical user interface (GUI) developed for subjective quality evaluation. *Each rater can see the three intensity projections along the x, y, and z directions visualized either with the conventional RSOM maximum intensity projection (MIP) or our tool for enhanced maximum intensity projection (eMIP). The rater can assign the scores 1 (=bad) to 4 (=perfect), undo the previous scan and return to it, mark the scan as "unsure" for later evaluation or mark the scan as "indefinite" if they do not want to label the given scan at all.*

Supplementary Tables

Supplementary Table 1. Mean and median baseline MOS values and corresponding improvements (Gain) achieved with eMIP are presented across different subject groups stratified by potential confounding factors. *The confounders include: RSOM system (one per clinical study), scan body location (leg or arm), skin pigmentation (Fitzpatrick types I/II for lighter skin, III/IV for darker skin), gender (female/male), body mass index (BMI), age, baseline image quality category (low, medium, high), and relative brightness between eMIP and MIP z-MIP images (Same brightness, MIP brighter and eMIP brighter). The number of scans for each group is reported in the second column. Confidence intervals (CI) for mean and median values are reported at a significance level of α = 0.01%.*

SUBGROUP	#Scans	MEAN			MEDIAN		
		Base	Gain	CI	Base	Gain	CI
System 1	949	1.885	0.254	[0.207, 0.301]	1.875	0.375	[0.250, 0.438]
System 2	776	1.986	0.247	[0.188, 0.306]	2.063	0.313	[0.250, 0.469]
Leg	224	1.688	0.264	[0.169, 0.360]	1.625	0.375	[0.178, 0.500]
Arm	136	1.805	0.311	[0.165, 0.457]	1.750	0.375	[0.125, 0.643]
Light skin	121	1.802	0.520	[0.315, 0.724]	1.625	0.938	[0.500, 1.500]
Darker skin	492	2.011	0.139	[0.081, 0.196]	2.063	0.219	[0.000, 0.375]
Female	435	2.003	0.225	[0.143, 0.307]	2.125	0.250	[0.125, 0.438]
Male	332	1.958	0.275	[0.188, 0.362]	2.000	0.375	[0.250, 0.563]
BMI < 29	389	1.978	0.282	[0.194, 0.370]	2.125	0.375	[0.250, 0.563]
BMI ≥ 29	378	1.990	0.210	[0.129, 0.290]	2.000	0.375	[0.164, 0.500]
Age < 65	336	2.041	0.252	[0.175, 0.328]	2.063	0.313	[0.188, 0.500]
Age ≥ 65	431	1.939	0.242	[0.154, 0.331]	2.000	0.438	[0.250, 0.625]
Low quality	449	1.129	0.315	[0.220, 0.410]	1.125	0.063	[0.000, 0.250]
Med quality	873	1.966	0.268	[0.220, 0.316]	2.000	0.250	[0.250, 0.375]
High quality	403	2.748	0.140	[0.088, 0.192]	2.750	0.125	[0.125, 0.250]
Same bright.	611	1.927	0.257	[0.197, 0.318]	2.000	0.250	[0.250, 0.438]
MIP brighter	474	1.936	0.244	[0.176, 0.312]	2.000	0.313	[0.250, 0.500]
eMIP brighter	1251	1.929	0.253	[0.209, 0.297]	1.938	0.313	[0.250, 0.500]
All	1725	1.931	0.250	[0.214, 0.287]	1.938	0.313	[0.250, 0.438]

Supplementary Table 2. General hyperparameters of the surface detection algorithm. *Quantile values α_i and multiplier values τ_i for "Find Surface Points". The last four alpha values in the table ($\alpha_{min,HF}$, $\alpha_{max,HF}$, $\alpha_{min,LF}$, $\alpha_{max,HF}$) are the quantiles for the upper and lower limits in "Adaptive Alpha". LF – low frequency reconstruction, HF – high frequency reconstruction, min – minimum, max – maximum, adpt. – adaptive alpha.*

sensitivity	α_1	α_2	$\tau_{1,HF}$	$\tau_{2,HF}$	$\tau_{1,LF}$	$\tau_{2,LF}$	$\tau_{adpt,HF}$	$\tau_{adpt,LF}$	$\alpha_{min,HF}$	$\alpha_{max,HF}$	$\alpha_{min,LF}$	$\alpha_{max,HF}$
1.75	0.625	0.925	0.5	1	1	1.25	1	1.25	0.15	0.975	0.5	0.975

Supplementary Table 3. Hyperparameters of the building block "Find Surface Points". *HF or LF indicates input reconstruction (high frequency reconstruction and low frequency reconstruction, respectively). Subscripts 1,2 and ADPT refers to the set of hyperparameters used (Supplementary Figure 3), for example HF_1 corresponds to α_1 and $\tau_{1,HF}$, and LF_{ADPT} corresponds to τ_{adpt} and α_{adpt} (see Methods). For a given iteration, the entries indicate the use of an input set. If 1: the corresponding set is used. If 2: the corresponding set is used, and points are duplicated. If "previous": surface points of the previous iteration are recycled. If "–": set is not used.*

ITERATION	HF_1	HF_2	LF_1	LF_2	HF_{ADPT}	LF_{ADPT}
**1**	–	1	1	1	–	–
**2**	1	–	previous	previous	–	–
**3**	1	–	2	–	–	–
**4**	–	–	–	–	1	1

Supplementary Table 4. Hyperparameters of the building block "Calculate Feasible Range". *Values for θ_{top} and θ_{bot} in Methods according to iteration, where t is the 90th percentile of the residuals (Eq. (19) in the main manuscript).*

ITERATION	$\boldsymbol{\theta_{top}}$	$\boldsymbol{\theta_{bot}}$
**1**	60	$-$
**2**	$30 + \mathrm{t}$	$-$
**3**	60	30

Supplementary Table 5. Hyperparameters of the building blocks "Exclude Outliers" & "Fit Surface Function". *The parameters $\sigma_{bot}, \sigma_{top}, \gamma_{bot}, \gamma_{top}$ and h_{abs} are as introduced in Methods. r is the 80th percentile of the absolute residuals. "TYPE" is the underling fitting algorithm, which was either RANSAC or a polynomial fit (Polyfit) based on the least square method. The parameters m (fast scanning axis) and n (slow scanning axis) are the possible polynomial degrees in the respective direction. In the preliminary "Polyfit" (0th iteration), no outlier exclusion is performed and a polynomial of degree one is fitted ($m = n = 1$).*

ITERATION	$\boldsymbol{\sigma_{top}}$	$\boldsymbol{\sigma_{bot}}$	$\boldsymbol{\gamma_{top}}$	$\boldsymbol{\gamma_{bot}}$	$\boldsymbol{h_{abs}}$	TYPE	$\boldsymbol{m}$	$\boldsymbol{n}$
**1**	∞	∞	∞	∞	30	RANSAC	1	1
**2**	∞	2	∞	0	30	Polyfit	1	1
**3**	2	2	0	0	60	Polyfit	1	1
**4**	2	1.5	2	1.5	60	Polyfit	$1-2$	$1-2$
**5**	2	3	1	1	60	Polyfit	$1-3$	$1-3$
**6**	∞	2	∞	1	$30 + r$	Polyfit	$2-3$	$1-3$
**7**	∞	0.75	∞	1.5	$30 + r$	Polyfit	$1-3$	$1-3$
**8**	3	1	1	1	60	RANSAC	$1-3$	$1-3$
**9**	∞	0.75	∞	0.75	∞	Polyfit	$1-3$	$1-3$
**10**	∞	0.75	∞	0.75	$30 + r$	Polyfit	$1-4$	$1-3$

Supplementary Table 6. Normalized percentile values across scans for threshold analysis. *Mean and standard deviation (std) of normalized percentile values computed across 200 scans, separately for high-frequency (HFR) and low-frequency (LFR) reconstructions. Percentile values were normalized to the 95th percentile of each scan. Percentile ranks between 80 and 97 yield stable, low-variance results, while higher ranks (≥98th) show increasing variability, particularly in HFR. These results support the robustness of the 95th percentile as a threshold for dynamic contrast adjustment.*

	**High Frequency Reconstruction**		**Low Frequency Reconstruction**	
**Percent Rank**	Mean	Std	Mean	Std
**80**	0.6425	0.0537	0.6827	0.0638
**90**	0.8188	0.0330	0.8427	0.0432
**95**	1	0	1	0
**97**	1.1393	0.0385	1.1163	0.0541
**98**	1.2548	0.0824	1.2098	0.1065
**99**	1.4627	0.1885	1.3693	0.2083
**99.8**	2.0031	0.5402	1.708	0.4500
**100**	3.8218	2.4062	2.2102	0.08743
